# Pleiotropic Effects of Rice Florigen Gene *RFT1* on the Amino Acid Content of Unmilled Rice

**DOI:** 10.3389/fgene.2020.00013

**Published:** 2020-01-31

**Authors:** Li-Hong Xie, Yu-Jun Zhu, Shao-Qing Tang, Xiang-Jin Wei, Zhong-Hua Sheng, Gui-Ai Jiao, Pei-Song Hu, Jie-Yun Zhuang

**Affiliations:** State Key Laboratory of Rice Biology/Chinese National Center for Rice Improvement, China National Rice Research Institute, Hangzhou, China

**Keywords:** amino acid content, heading date, near isogenic line, pleiotropic effect, quantitative trait locus, *Oryza sativa* L.

## Abstract

In rice, the contents of protein and amino acids are the major parameters of nutritional quality. Co-localization of quantitative trait loci (QTLs) for heading date and protein content were reported, but pleiotropism of heading-date genes on protein contents has not been investigated. Here, we reported that rice florigen gene *RFT1* plays an important role in controlling amino acid contents of rice grain. Firstly, 73 QTLs for the contents of 17 amino acids in unmilled rice were detected using recombinant inbred lines (RILs) of the *indica* rice cross Zhenshan 97 (ZS97)/Milyang 46 (MY46). Then, the effect of the largest cluster consisting of 14 QTLs, located in proximity to the rice florigen genes *RFT1* and *Hd3a*, was validated using three populations consisting of near isogenic lines (NILs) that only segregated a region covering the target QTL. The first and second NIL populations were derived from a residual heterozygote identified from the ZS97/MY46 RIL population, consisting of homozygous lines that were only segregated in a 29.9-kb region covering the two florigen genes and a 1.7-kb region for *RFT1*, respectively. The third NIL population was segregated for the *RFT1* ^ZS97^ transgene in the background of *japonica* rice cultivar Zhonghua 11. In all the three NIL populations, *RFT1* was shown to have a strong effect on the contents of most amino acids, with the ZS97 allele always having the reducing effects. By comparing QTLs for amino acid contents detected in the ZS97/MY46 RIL population and genes/QTLs previously identified for heading date difference between ZS97 and MY46, possible pleiotropism on amino acid contents was also shown for other key heading-date genes including *Hd1*, *Ghd7*, and *OsGI*.

## Introduction

Rice sustains about half of the world's population, providing a source of energy and protein. Protein content (PC) of the rice grain is influenced by both genotype and growing environment. The PC values in the un-milled (brown) and milled rice of a large collection of rice cultivars were found to range as 5.6−11.2 and 6.0−15.7%, respectively, with a high correlation coefficient of 0.96 between the two measurements (Chen et al., 2006). In addition to the quantity of total protein, contribution of different amino acids is also an important factor determining the nutritional value of rice grain (Wang et al., 2008). An understanding of the genetic basis underlying the variation of the total grain protein content and the contribution of individual amino acids has the potential to facilitate the breeding of rice cultivars having nutritionally superior grain.

A number of attempts have been made to identify the genetic architecture of the spectrum of grain amino acids in rice by means of quantitative trait locus (QTL) analysis. Using recombinant inbred lines (RILs) derived from a cross between rice cultivars Zhenshan 97 (ZS97) and Nanyangzhan, 18 QTL clusters for 19 components of the amino acid content (AAC) in milled rice were identified (Wang et al., 2008). In two other RIL populations derived from crosses using ZS97 as the female parent, ZS97/Minghui 63 (Lu et al., 2009) and ZS97/Delong 208 (Zhong et al., 2011), 5 and 29 QTL regions for 10 and 17 components of AAC in milled rice were detected, respectively. Many more studies examined the total protein content without analysis on individual amino acids. A region covering the *Wx* locus on the short arm of chromosome 6 was frequently found to be associated with PC (Tan et al., 2001; Aluko et al., 2004; Wada et al., 2006; Yu et al., 2009; Kashiwagi and Munakata, 2018), but its influence on AAC was only occasionally observed (Wang et al., 2008; Lu et al., 2009; Zhong et al., 2011). Whether the *Wx* gene itself or other linked genes are involved in the genetic control of PC and AAC remains to be determined.

In rice, the short arm of chromosome 6 is a region harboring multiple genes that play critical roles in the regulation of heading date (HD), including *Hd1*, *Hd3a*, *RFT1*, and *Hd17*/*Hd3b* (Hori et al., 2016). In a number of segregating populations that derived from intra-subspecies or inter-species crosses, negative correlation between HD and PC was observed (Wada et al., 2006; Kwon et al., 2011; Yun et al., 2016), which could be partially ascribed to a QTL region on the short arm of chromosome 6 that affected both HD and PC with opposite allelic directions (Wada et al., 2006; Yun et al., 2016). These results suggest that one or more heading-date genes located in this region may have pleiotropic effects on the contents of proteins and amino acids. In the present study, QTL analysis for 17 components of AAC in unmilled rice was performed using the ZS97/Milyang 46 (MY46) RIL population, followed by the validation of a QTL cluster on the short arm of chromosome 6 using three populations of near isogenic lines (NILs) in either *indica* or *japonica* backgrounds. A total of 73 QTLs were detected, and the *RFT1* gene was found to have a strong and stable pleiotropic effect on AAC.

## Material and Methods

### Plant Materials

Four segregating populations of rice (*Oryza sativa* L.) were used in this study. One was a primary mapping population consisting of 247 RILs developed from a cross between *indica* rice cultivars ZS97 and MY46. The other three were NIL populations segregating a region involving the *RFT1* gene in an isogenic background, all of which have been reported by Zhu et al. (2017). Two of the NIL populations, namely TF6-15 and R1, were derived from a residual heterozygote (RH) of ZS97/MY46. An F_10_ plant was selected, which was heterozygous in a 29.9-kb region covering the *RFT1* and *Hd3a* loci and homozygous in other regions. The S_1_ plants were assayed with DNA markers located in the segregating region. Homozygous plants were selfed to produce NILs. The TF6-15 population was established, consisting of 10 lines of ZS97 homozygotes and 10 lines of MY46 homozygotes differing in the 29.9 kb region. New RHs were identified from heterozygous progeny of the F_10_ plant. An F_14_ plant that was heterozygous at the *RFT1* locus only was selected. The R1 population was constructed, comprising 20 lines of ZS97 homozygotes and 20 lines of MY46 homozygotes differing for the *RFT1* gene only. The remaining NIL population consisted of 28 homozygous transgenic lines in the genetic background of *japonica* rice cultivar Zhonghua 11 (ZH11), of which 14 lines carried the *RFT1* ^ZS97^ transgene and the others carried no transgene.

### Field Experiments

The four populations were planted in the single-cropping rice season at the China National Rice Research Institute in Hangzhou, Zhejiang, China. The RIL population was raised in 2015, 2016, and 2017, and the other three populations were grown in 2017 only. Each line was represented by 12 plants per row, with an inter-plant spacing of 16.7 cm and an inter-row spacing of 26.7 cm. The RILs were grown without replication, while the other populations were represented by two replicates. Plants were managed using standard agricultural practice.

### Determination of Amino Acid Content

Grain was bulked from the middle ten plants of each row and dried to a moisture content of ~12%. De-hulling was achieved using THU-35A testing husker (Satake Engineering Co. Ltd., Hiroshima, Japan). The dehusked grain was ground using a Cyclotec 1093 Sample Mill (Tecator, Hoganas, Sweden) and the resulting flour was passed through a 0.42 mm sieve. A 250 mg batch of each flour sample was sealed in a vial containing 10 ml 6.0 M HCl and held at 110°C for 24 h. The resulting hydrolysate was diluted to 50 ml with deionized water and filtered. A 0.2 ml aliquot of the filtrate was transferred to a 2 ml tube and evaporated down to ~0.1 ml by bubbling nitrogen gas. After the addition of 2 ml 20 mM HCl, the solution was passed through a 0.2 μm Acrodisc membrane (Pall Corp., Port Washington, NY, USA). The amino acid content of each sample was acquired using a L8900 amino acid auto analyzer (Hitachi, High-Technology Corporation, Tokyo, Japan). Percentage contributions of each of the 17 amino acids were obtained using Ezchrom Elite software (High-Technology Corporation, Tokyo, Japan).

The amino acids studied were aspartic acid (Asp), threonine (Thr), serine (Ser), glutamic acid (Glu), glycine (Gly), alanine (Ala), cystine (Cys), valine (Val), methionine (Met), isoleucine (Ile), leucine (Leu), tyrosine (Tyr), phenylalanine (Phe), lysine (Lys), histidine (His), arginine (Arg), and proline (Pro). Each sample was analyzed in triplicate. Every analytical batch included a blank (20 mM HCl only) and a GBW (E) 100010 reference sample (Chinese National Research Center for Certified Reference Materials, Beijing, China), except that the reference samples for Asp and Cys were not available. Concentrations in the standard samples of Ile, Leu, Tyr, Phe, Lys, His, Arg, Thr, Ser, Glu, Pro, Gly, Ala, Met, and Val measured based on six replicates were 0.38 ± 0.060, 0.74 ± 0.040, 0.30 ± 0.020, 0.52 ± 0.068, 0.28 ± 0.012, 0.22 ± 0.012, 0.49 ± 0.048, 0.30 ± 0.014, 0.49 ± 0.014, 3.28 ± 0.18, 0.95 ± 0.010, 0.40 ± 0.048, 0.503 ± 0.026, 0.165 ± 0.046, and 0.50 ± 0.024%, respectively, matching well with the Certified Reference Material's values. The day-to-day reproducibility of the assay checked over three days was satisfactory, having the relative standard deviation lower than 5.0% for all the fifteen amino acids.

### Quantitative Trait Locus Mapping

Genetic map of the ZS97/MY46 RIL population was previously constructed, consisting of 256 markers and spanning 1,814.7 cM (Wang et al., 2017b). This map was applied for QTL analysis using composite interval mapping (CIM) and multiple interval mapping (MIM) in Windows QTL Cartographer v2.5 (Wang et al., 2011). A candidate QTL was identified with CIM using a threshold of logarithm of odds (LOD) > 2.0 and then evaluated with MIM using the Bayesian Information Criterion c (n) = ln (n). A putative QTL was claimed if it satisfied both criteria. QTLs were designated as proposed by McCouch and CGSNL (2008). Two-way analysis of variance (ANOVA) was conducted to test the differences between the two genotypic groups in each of the three NIL populations, using a general linear model (GLM) of the SAS Program as described by Dai et al. (2008).

## Results

### Variation of Amino Acid Contents in the ZS97/MY46 Recombinant Inbred Line Population

Variation of the 17 components of AAC in unmilled rice of the ZS97/MY46 RIL population is summarized in **Table 1**. There was a strong evidence for transgressive segregation in both directions for all the amino acids except His of which the contents of the RILs were all lower than the high-parental value in 2016. It was also shown that differences between the two parental lines varied greatly across the 3 years. Among the contents of the 17 amino acids, ZS97 was found to be the high-value parent for two amino acids only (Phe and His) in 2015, but ZS97 was the high-value parent for seven amino acids (Ser, Glu, Leu, Tyr, Phe, Lys, and His) in 2016 and for 14 amino acids (Asp, Thr, Ser, Glu, Gly, Ala, Val, Ile, Leu, Tyr, Phe, Lys, His, and Arg) in 2017.

**Table 1:** Phenotype performance of 17 components of amino acid content in the ZS97/MY46 recombinant inbred line (RIL) population.

Trait^a^		Parental mean		RIL Population				
		**ZS97**	**MY46**	**Mean**	**Range**	**SD**	**Skewness**	**Kurtosis**
Asp	2015	0.848	1.048	0.904	0.632–1.302	0.101	0.359	0.794
	2016	0.819	0.851	0.849	0.629–1.012	0.072	−0.053	−0.050
	2017	1.013	0.904	0.859	0.424–1.163	0.096	−0.341	2.049
Thr	2015	0.354	0.403	0.372	0.265–0.520	0.042	−0.087	0.385
	2016	0.341	0.357	0.359	0.273–0.433	0.029	0.009	0.127
	2017	0.427	0.386	0.369	0.243–0.490	0.034	0.133	0.877
Ser	2015	0.500	0.544	0.517	0.353–0.741	0.062	0.087	0.369
	2016	0.490	0.481	0.482	0.367–0.585	0.039	−0.080	−0.020
	2017	0.540	0.481	0.467	0.295–0.637	0.046	0.141	1.037
Glu	2015	1.782	1.877	1.812	1.181–2.451	0.225	0.034	0.099
	2016	1.757	1.683	1.644	1.203–1.990	0.161	−0.094	−0.565
	2017	1.741	1.554	1.619	1.075–2.242	0.161	0.358	1.111
Gly	2015	0.233	0.273	0.235	0.170–0.360	0.027	0.0004	0.372
	2016	0.236	0.247	0.216	0.156–0.262	0.018	−0.249	0.194
	2017	0.218	0.199	0.269	0.161–0.585	0.128	1.344	0.005
Ala	2015	0.218	0.249	0.247	0.155–0.331	0.027	0.531	1.183
	2016	0.221	0.221	0.240	0.183–0.354	0.020	0.874	4.783
	2017	0.218	0.199	0.422	0.171–0.665	0.122	−0.905	−0.520
Cys	2015	0.396	0.522	0.561	0.245–0.862	0.105	0.058	0.347
	2016	0.556	0.702	0.507	0.125–0.849	0.253	−0.542	−1.530
	2017	0.268	0.272	0.302	0.213–0.423	0.035	0.730	0.580
Val	2015	0.420	0.483	0.458	0.304–0.618	0.062	0.068	−0.351
	2016	0.425	0.482	0.441	0.331–0.542	0.038	0.084	−0.249
	2017	0.439	0.406	0.422	0.296–0.548	0.041	0.354	0.587
Met	2015	0.030	0.068	0.053	0.000–0.203	0.031	1.086	2.576
	2016	0.033	0.110	0.060	0.005–0.172	0.029	0.640	0.571
	2017	0.072	0.103	0.029	0.000–0.121	0.031	0.852	−0.298
Ile	2015	0.256	0.308	0.287	0.198–0.440	0.040	0.788	1.229
	2016	0.257	0.299	0.290	0.220–0.356	0.027	−0.036	−0.205
	2017	0.275	0.248	0.269	0.184–0.363	0.028	0.464	0.628
Leu	2015	0.734	0.815	0.762	0.534–1.085	0.086	0.452	0.452
	2016	0.743	0.724	0.703	0.502–0.856	0.065	−0.148	−0.121
	2017	0.670	0.615	0.658	0.420–0.927	0.071	0.449	0.958
Tyr	2015	0.352	0.353	0.273	0.133–0.415	0.075	−0.117	−1.103
	2016	0.359	0.282	0.272	0.090–0.362	0.033	0.010	0.407
	2017	0.184	0.175	0.211	0.144–0.351	0.034	0.950	1.460
Phe	2015	0.592	0.578	0.546	0.367–0.802	0.077	0.548	0.711
	2016	0.598	0.491	0.488	0.336–0.629	0.049	0.023	0.298
	2017	0.411	0.396	0.434	0.292–0.670	0.060	0.784	1.660
Lys	2015	0.369	0.378	0.326	0.216–0.471	0.052	0.652	0.129
	2016	0.370	0.356	0.340	0.258–0.419	0.030	0.251	0.192
	2017	0.306	0.293	0.319	0.250–0.433	0.034	0.641	0.712
His	2015	0.305	0.298	0.237	0.139–0.393	0.048	1.236	1.654
	2016	0.304	0.222	0.221	0.156–0.288	0.023	0.290	0.235
	2017	0.235	0.215	0.225	0.180–0.292	0.021	0.435	0.041
Arg	2015	0.617	0.769	0.658	0.421–0.993	0.077	0.480	1.355
	2016	0.627	0.659	0.631	0.458–0.774	0.057	0.108	0.276
	2017	0.657	0.559	0.585	0.443–0.782	0.065	0.323	−0.159
Pro	2015	0.402	0.468	0.274	0.098–0.499	0.093	0.445	−0.740
	2016	0.189	0.234	0.269	0.006–0.536	0.076	0.730	1.248
	2017	0.216	0.226	0.253	0.020–0.539	0.094	0.931	−0.025

a Contents of the amino acids are presented as % in unmilled rice. Asp, aspartic acid; Thr, threonine; Ser, serine; Glu, glutamic acid; Gly, glycine; Ala, alanine; Cys, cystine; Val, valine; Met, methionine; Ile, iIsoleucine; Leu, leucine; Tyr, tyrosine; Phe, phenylalanine; Lys, lysine; His, histidine; Arg, arginine and Pro, proline.

Pearson correlation coefficients between the 17 components of AAC were calculated using mean values over 3 years and data of each year. Family error rates were controlled by dividing the *P* value of 0.05 by 17, thus a threshold of *P* < 0.003 was used for declaring a significant correlation. It was found that a large majority of the correlations were positively significant. Of the 136 estimates produced from the mean values, 114 were positively significant, 4 were negatively significant, and 18 were non-significant (**Table 2**). The four negative correlations occurred between Ala and Gly, Cys, Met, and Pro. The 18 non-significant correlations included nine between Ala and others (Ser, Glu, Val, Ile, Leu, Tyr, Phe, Lys, and Arg), 8 between Cys and others (Asp, Thr, Gly, Val, Met, Lys, His, and Pro), and 1 between Tyr and Met. Common occurrence of significantly positive correlations between different AAC components were also observed when data of each year were used (**Supplementary Table S1**). In 2015, 101 correlations were positively significant and the other 35 were non-significant. In 2016, 122 correlations were positively significant and the other 14 were non-significant. In 2017, 118 correlations were positively significant, three were negatively significant, and 15 were non-significant.

**Table 2:** Pearson correlation coefficients between 17 components of AAC in the ZS97/MY46 recombinant inbred line (RIL) population.

Trait	Asp	Thr	Ser	Glu	Ala	Gly	Cys	Val	Met	Ile	Leu	Tyr	Phe	Lys	His	Arg
Thr	0.96^*^															
Ser	0.94^*^	0.94^*^														
Glu	0.88^*^	0.88^*^	0.94^*^													
Ala	0.23^*^	0.30^*^	0.19	0.17												
Gly	0.32^*^	0.30^*^	0.31^*^	0.34^*^	−0.62^*^											
Cys	0.13	0.06	0.20^*^	0.25^*^	−0.22^*^	0.04										
Val	0.86^*^	0.87^*^	0.91^*^	0.92^*^	0.11	0.38^*^	0.17									
Met	0.30^*^	0.29^*^	0.37^*^	0.36^*^	−0.24^*^	0.37^*^	0.16	0.48^*^								
Ile	0.84^*^	0.85^*^	0.88^*^	0.90^*^	0.15	0.33^*^	0.19^*^	0.95^*^	0.43^*^							
Leu	0.88^*^	0.86^*^	0.94^*^	0.95^*^	0.15	0.31^*^	0.27^*^	0.92^*^	0.37^*^	0.94^*^						
Tyr	0.42^*^	0.33^*^	0.44^*^	0.44^*^	−0.10	0.20^*^	0.45^*^	0.39^*^	0.15	0.47^*^	0.57^*^					
Phe	0.77^*^	0.75^*^	0.86^*^	0.87^*^	0.04	0.33^*^	0.30^*^	0.86^*^	0.40^*^	0.86^*^	0.93^*^	0.58^*^				
Lys	0.79^*^	0.78^*^	0.78^*^	0.73^*^	0.14	0.29^*^	0.13	0.74^*^	0.27^*^	0.77^*^	0.78^*^	0.51^*^	0.80^*^			
His	0.75^*^	0.77^*^	0.78^*^	0.75^*^	0.25^*^	0.22^*^	0.08	0.73^*^	0.25^*^	0.72^*^	0.76^*^	0.47^*^	0.76^*^	0.84^*^		
Arg	0.88^*^	0.87^*^	0.93^*^	0.90^*^	0.13	0.33^*^	0.24^*^	0.91^*^	0.39^*^	0.89^*^	0.93^*^	0.54^*^	0.87^*^	0.80^*^	0.81^*^	
Pro	0.40^*^	0.36^*^	0.38^*^	0.42^*^	−0.37^*^	0.66^*^	0.16	0.37^*^	0.24^*^	0.38^*^	0.41^*^	0.47^*^	0.41^*^	0.41^*^	0.33^*^	0.37^*^

^*^P < 0.003. The correlation coefficients were calculated based on mean values over 3 years.

### Quantitative Trait Loci for Amino Acid Contents Detected in the ZS97/MY46 Recombinant Inbred Line Population

**Figure 1:**
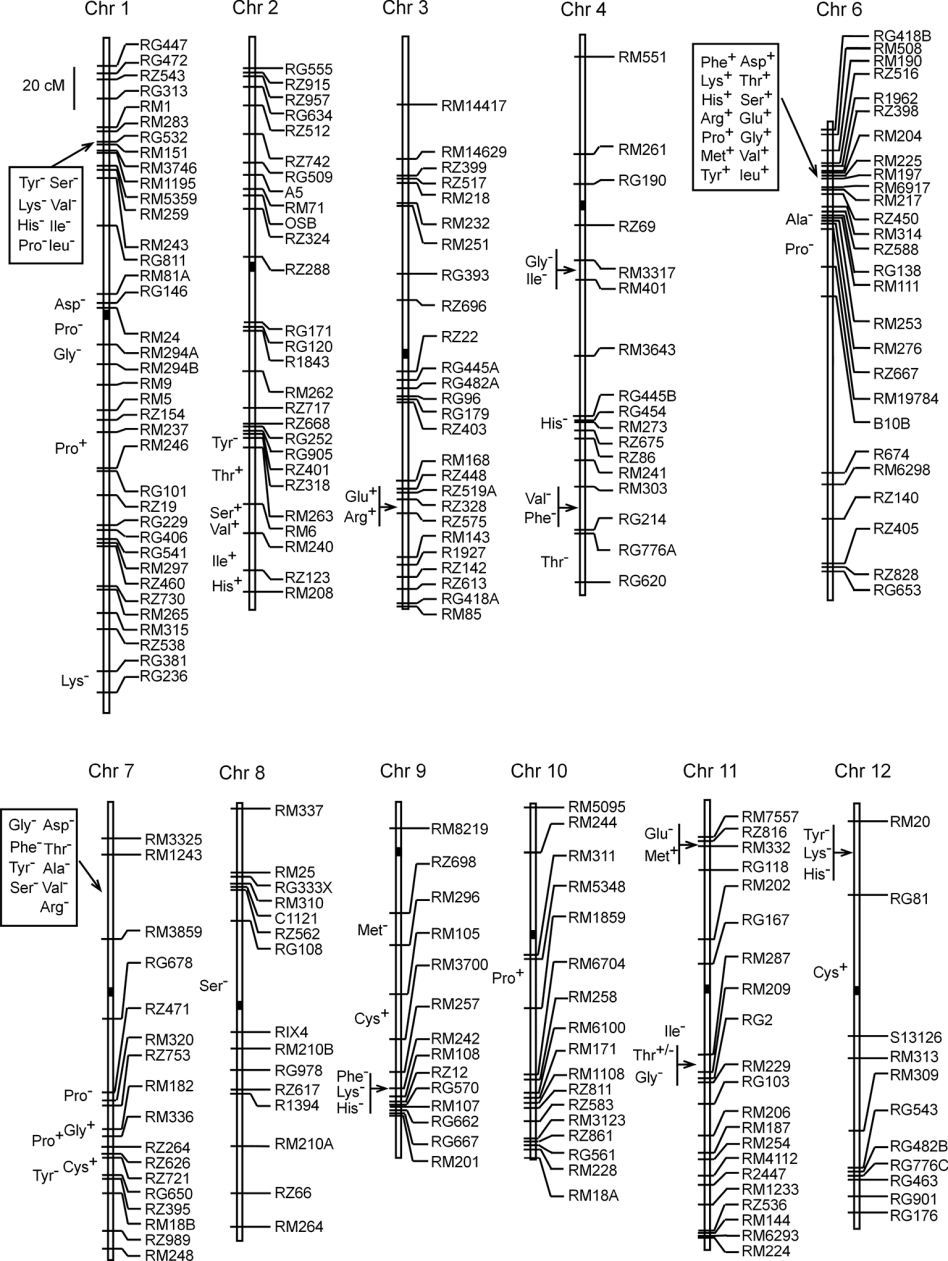
Chromosomal locations of quantitative trait loci for 17 components of amino acid contents detected in the recombinant inbred line population of Zhenshan 97/Milyang 46.

A total of 73 QTLs were detected based on 3-year's data of the 17 components of AAC in the ZS97/MY46 RIL population (**Supplementary Table S2**). Of these QTLs, seven were identified in all the 3 years, eight were found in 2 years, and the others were detected in 1 year only. The number of QTLs detected for each amino acid ranged from two to eight, with the proportion of the variance explained (*R^2^*) by a single QTL ranging from 2.2 to 35.9%. These QTL were distributed over all the 12 rice chromosomes except chromosome 5 (**Figure 1**). Most of them were located in cluster, with chromosomes 1, 6, and 7 harboring the highest number of loci. Of the 15 QTLs detected in 2 or 3 years, 12, 2, and 1 were located in chromosomes 6, 7, and 11, respectively. Except *qThr11*, allelic directions of these QTLs all remained consistent across different years.

Fourteen QTLs were located in the RM190–RM6917 region on the short arm of chromosome 6, forming the largest cluster in terms of QTL number. Included were seven QTLs detected in three years (*qAsp6*, *qSer6*, *qGly6*, *qLeu6*, *qPhe6*, *qHis6,* and *qArg6*), five QTLs detected in 2 years (*qThr6*, *qGlu6*, *qVal6*, *qMet6,* and *qTyr6*), and two QTLs detected in 1 year (*qLys6* and *qPro6.1*). Enhancing alleles of these QTLs were all derived from the male parent MY46, with *qGly6* having the highest *R^2^* of 33.9%. Two other QTLs (*qAla6* and *qPro6.2*) were detected in nearby intervals RM253–RM276 and RZ667–RM19784, respectively, of which the enhancing alleles were both derived from the female parent ZS97. Altogether, 16 QTLs were detected on chromosome 6.

The second largest cluster consisting of nine QTLs was located in the RM3325–RM3859 region on the short arm of chromosome 7. Included were two QTLs detected in 2 years (*qAsp7* and *qArg7*) and seven QTLs detected in 1 year (*qThr7*, *qSer7*, *qGly7.1*, *qAla7*, *qVal7*, *qTyr7.1*, and *qPhe7*). Enhancing alleles of these QTLs were all derived from ZS97, with *qAsp7* having the highest *R^2^* of 19.8%. Five other QTLs were clustered in the RZ471–RZ395 region on the long arm of this chromosome. The enhancing alleles were derived from ZS97 at *qTyr7.2* and *qPro7.1*, and from MY46 at *qGly7.2*, *qCys7*, and *qPro7.2*. The five QTLs had high *R^2^* ranging from 10.7 to 35.9%. Altogether, 14 QTLs were detected on chromosome 7.

The third largest cluster consisting eight QTLs (*qSer1*, *qVal1*, *qIle1*, *qLeu1*, *qTyr1*, *qLys1.1*, *qHis1*, and *qPro1.1*) was located in the RM283–RM3746 region on the short arm of chromosome 1. Enhancing alleles of these QTLs were all derived from ZS97, with *qLeu1* having the highest *R^2^* of 15.0%. Three other QTLs (*qAsp1*, *qGly1*, and *qPro1.2*) were located in the pericentromeric region of chromosome 1. They had high *R^2^* ranging from 17.0 to 32.8%, and the enhancing alleles were all derived from ZS97. Two sparsely-distributed QTLs (*qPro1.3* and *qLys1.2*) were located in lower regions of the long arm. Altogether, 13 QTLs were detected on chromosome 1.

Among the remaining 30 QTLs, two single QTL were located on chromosomes 8 and 10, respectively, and the others were distributed on chromosomes 2, 3, 4, 9, 11, and 12 with 2–6 QTLs per chromosome. The six QTLs on chromosome 2 were all located in the lower region of the long arm; the two QTLs on chromosome 3 were tightly linked; the six QTLs on chromosome 4 involved two pairs of tightly-linked QTLs with two nearby QTLs; the five QTLs on chromosome 9 may be viewed as one cluster and two single QTL; the five QTLs on chromosome 11 was separated into two clusters; and the four QTLs on chromosome 12 included one cluster and one single QTL.

### Effect of *RFT1* on Amino Acid Contents Detected Between NIL^ZS97^ and NIL^MY46^

As described above, the largest QTL cluster for the 17 components of AAC detected in the ZS97/MY46 RIL population was located in the RM190–RM6917 region on the short arm of chromosome 6. This region covered the two florigen genes of rice, *RFT1* and *Hd3a* (Hori et al., 2016), suggesting a possible involvement of *RFT1* and/or *Hd3a* in controlling AAC of rice grain. This assumption was firstly tested using the NIL population TF6-15 segregating a 29.9-kb interval covering both *RFT1* and *Hd3a*. Significant differences (*P* < 0.05) between the 10 homozygous lines of NIL^ZS97^ and 10 homozygous lines of NIL^MY46^ were detected on 15 of the 17 components of AAC (**Table 3**). The *R^2^* for individual components ranged from 16.7 to 61.2%. The enhancing alleles were all derived from MY46, which is in agreement with the effects detected in the ZS97/MY46 RIL population.

**Table 3:** Effects of the *RFT1*-*Hd3a* region on 17 components of amino acid content (AAC) detected in the TF6-15 population.

Trait	Phenotype (mean±SE)^a^		*P*	*A^b^*	*R* ^2^ (%)*^c^*
	NIL^ZS97^	NIL^MY46^			
Asp	0.721±0.006	0.762±0.010	0.0007	0.020	39.8
Thr	0.308± 0.003	0.326±0.004	0.0009	0.009	37.6
Ser	0.427± 0.004	0.452±0.005	0.0005	0.013	38.8
Glu	1.543±0.008	1.626±0.015	0.0003	0.042	42.0
Gly	0.183± 0.001	0.193±0.001	0.0002	0.005	50.0
Ala	0.421±0.004	0.447±0.007	<0.0001	0.013	46.3
Cys	0.273±0.003	0.286±0.006	0.0034	0.006	28.9
Val	0.331±0.005	0.359±0.008	0.0033	0.014	26.3
Met	0.047±0.003	0.051±0.005	0.4121		
Ile	0.202±0.004	0.221±0.005	0.0063	0.009	22.6
Leu	0.577±0.004	0.614±0.006	<0.0001	0.019	47.1
Tyr	0.165±0.002	0.177±0.004	0.0295	0.006	16.7
Phe	0.382±0.003	0.413±0.005	<0.0001	0.015	54.9
Lys	0.231±0.003	0.250±0.005	<0.0001	0.010	52.1
His	0.167± 0.002	0.180±0.003	<0.0001	0.007	51.3
Arg	0.499±0.003	0.544±0.007	<0.0001	0.023	61.2
Pro	0.204±0.011	0.224±0.005	0.0534		

a NIL^ZS97^ and NIL^MY46^ are near isogenic lines carrying homozygous alleles from ZS97 and MY46, respectively.

b Additive effect of replacing a ZS97 allele with a MY46 allele.

c Proportion of phenotypic variance explained by the QTL effect. *R^2^* = V_G_/V_P_ ×100, in which V_G_ is the variance between the two genotypic groups, and V_P_ the phenotypic variance.

Then, QTL analysis was performed using the NIL population R1 that was homozygous at the *Hd3a* locus but segregated for the *RFT1* gene. Significant differences (*P* < 0.05) between the 20 homozygous lines of NIL^ZS97^ and 20 homozygous lines of NIL^MY46^ were detected on 15 of the 17 components of AAC (**Table 4**). The *R^2^* for individual components ranged from 9.5 to 63.2%. Again, the enhancing alleles were all derived from MY46. It is also noted that the two components showing no significant difference between NIL^ZS97^ and NIL^MY46^ were commonly found to be Met and Pro in the TF6-15 and R1 populations. These results indicate that the *RFT1* gene has a strong and stable effect on most components of AAC in unmilled rice.

**Table 4:** Effects of the *RFT1* gene on 17 components of amino acid content (AAC) detected in the R1 population.

Trait	Phenotype (mean ± SE) ^a^		*P*	*A^b^*	*R^2^*(%)*^c^*
	**NIL^ZS97^**	**NIL^MY46^**			
Asp	0.733 ± 0.006	0.819 ± 0.007	<0.0001	0.043	58.7
Thr	0.328 ± 0.002	0.362 ± 0.002	<0.0001	0.017	62.8
Ser	0.432 ± 0.003	0.480 ± 0.004	<0.0001	0.024	63.2
Glu	1.495 ± 0.012	1.660 ± 0.012	<0.0001	0.083	59.8
Gly	0.173 ± 0.001	0.192 ± 0.002	<0.0001	0.010	56.5
Ala	0.440 ± 0.004	0.485 ± 0.003	<0.0001	0.023	53.6
Cys	0.321 ± 0.003	0.334 ± 0.004	0.0009	0.006	10.4
Val	0.377 ± 0.003	0.415 ± 0.004	<0.0001	0.019	46.6
Met	0.066 ± 0.002	0.067 ± 0.002	0.6776		
Ile	0.238 ± 0.002	0.261 ± 0.002	<0.0001	0.012	45.4
Leu	0.595 ± 0.005	0.662 ± 0.005	<0.0001	0.033	58.2
Tyr	0.202 ± 0.003	0.217 ± 0.005	0.0026	0.007	9.5
Phe	0.392 ± 0.004	0.445 ± 0.004	<0.0001	0.026	57.1
Lys	0.266 ± 0.003	0.292 ± 0.002	<0.0001	0.013	42.6
His	0.181 ± 0.002	0.202 ± 0.002	<0.0001	0.011	55.5
Arg	0.500 ± 0.005	0.570 ± 0.006	<0.0001	0.035	52.5
Pro	0.185 ± 0.006	0.202 ± 0.006	0.0582		

a NIL^ZS97^ and NIL^MY46^ are near isogenic lines carrying homozygous alleles from ZS97 and MY46, respectively.

b Additive effect of replacing a ZS97 allele with a MY46 allele.

c Proportion of phenotypic variance explained by the QTL effect. *R^2^* = V_G_/V_P_ ×100, in which V_G_ is the variance between the two genotypic groups, and V_P_ the phenotypic variance.

### Effect of the *RFT1* ^ZS97^ Transgene on Amino Acid Contents in a *Japonica* Rice Background

The effect of *RFT1* on AAC in unmilled rice was further tested using a transgenic population segregating the *RFT1* ^ZS97^ transgene in the genetic background of *japonica* cultivar ZH11. Significant differences (*P* < 0.05) between the 14 lines of NIL^ZS97^ carrying homozygous transgenes and 14 lines of NIL^ZH11^ carrying no transgene were detected on 13 of the 17 components of AAC, with *R^2^* ranging from 8.1 to 38.0% (**Table 5**). Integration of the *RFT1* ^ZS97^ transgene into the genome of ZH11 reduced the contents of the amino acids. In addition, the two components showing no significant difference between NIL^ZS97^ and NIL^MY46^ in the TF6-15 and R1 populations, Met and Pro, were included in the four components having no significant difference between NIL^ZS97^ and NIL^ZH11^ in the transgenic population. These results indicate that the effects of *RFT1* on AAC of unmilled rice are consistent in the genetic background of different subspecies of Asian cultivated rice.

**Table 5:** Effects of *RFT1* ^ZS9^ *^7^* transgene on 17 components of amino acid content (AAC).

Trait	Phenotype (mean ± SE)^a^		*P*	*A^b^*	*R^2^* (%)^c^
	**NIL^ZS97^**	**NIL^ZH11^**			
Asp	0.745 ± 0.009	0.789 ± 0.008	<0.0001	−0.022	28.1
Thr	0.315 ± 0.004	0.328 ± 0.003	<0.0001	−0.006	14.8
Ser	0.423 ± 0.004	0.440 ± 0.004	<0.0001	−0.008	19.6
Glu	1.380 ± 0.017	1.423 ± 0.014	0.0191	−0.021	8.1
Gly	0.184 ± 0.002	0.192 ± 0.002	0.0002	−0.004	17.2
Ala	0.441 ± 0.005	0.457 ± 0.005	0.0005	−0.008	13.9
Cys	0.264 ± 0.002	0.280 ± 0.003	0.0014	−0.008	22.7
Val	0.369 ± 0.005	0.378 ± 0.005	0.1036		
Met	0.075 ± 0.003	0.086 ± 0.003	0.0975		
Ile	0.221 ± 0.004	0.226 ± 0.003	0.1427		
Leu	0.558 ± 0.006	0.574 ± 0.005	0.0008	−0.008	11.5
Tyr	0.166 ± 0.002	0.175 ± 0.003	0.0007	−0.005	13.8
Phe	0.380 ± 0.004	0.397 ± 0.004	0.0003	−0.009	17.8
Lys	0.273 ± 0.003	0.292 ± 0.003	<0.0001	−0.010	36.0
His	0.187 ± 0.002	0.199 ± 0.001	<0.0001	−0.006	38.0
Arg	0.533 ± 0.006	0.563 ± 0.005	<0.0001	−0.015	32.7
Pro	0.182 ± 0.005	0.183 ± 0.006	0.9528		

a NIL^ZS97^ and NIL^ZH11^ are T_3_ transgenic lines carrying *RFT1* ^ZS97^ homozygous transgene and no transgene in the genetic background of ZH11, respectively.

b Additive effect of replacing a ZH11 allele with a ZS97 allele.

c Proportion of phenotypic variance explained by the QTL effect. *R^2^* = V_G_/V_P_ ×100, in which V_G_ is the variance between the two genotypic groups, and V_P_ the phenotypic variance.

## Discussion

Heading date, grain yield, and grain quality are three basic traits influencing the commercial utilization of a rice cultivar. The regional and seasonal adaptation is mostly determined by heading date, the productivity is measured by grain yield, and whether the product can meet the demand of end-users is mainly characterized by grain quality. A number of key genes for flowering regulation in rice have been found to play important roles in the genetic control of yield traits, including *Ghd7* (Xue et al., 2008; Weng et al., 2014), *DTH8*/*Ghd8* (Wei et al., 2010; Yan et al., 2011), *Hd1* (Zhang et al., 2012; Zhang et al., 2015; Ye et al., 2018), *Ghd7.1* (Yan et al., 2013), and *RFT1* (Zhu et al., 2017). On the other hand, no study has been reported for the pleiotropic effects of heading-date genes on grain quality in rice. Among traits in the four primary categories of rice grain quality (i.e., milling, appearance, eating and cooking, and nutritional qualities), PC and AAC are the major parameters of nutritional quality (Wang et al., 2008; Wang et al., 2017a). In the present study, a total of 73 QTLs for AAC of unmilled rice were detected using the ZS97/MY46 RIL population, and the largest QTL cluster was validated to be responsible by the *RFT1* gene on the short arm of chromosome 6. It is also evident that the effect of *RFT1* is consistent across different genetic backgrounds. In accordance with the common occurrence of significantly positive correlations between different components of AAC (**Table 2**; **Supplementary Table S1**), most of the QTLs were located in cluster and different QTL in a given region usually had the same allelic direction (**Figure 1**; **Supplementary Table S2**).

*RFT1* protein is the florigen for promoting the flowering of rice under long-day (LD) conditions (Tsuji et al., 2011). As compared to the ZS97 allele of *RFT1*, the MY46 and ZH11 alleles were shown to promote heading in rice populations grown under natural LD conditions in Hangzhou (Zhu et al., 2017). Replacing a ZS97 allele by a MY46 allele in the R1 population promoted flowering by 11.63 to 15.61 days over 3 years; and replacing a ZS97 allele by a ZH11 allele promoted flowering by 3.91 and 6.12 days in the transgenic population in 2 years. In the present study, replacement of the ZS97 allele of *RFT1* with the MY46 and ZH11 alleles resulted in increasing the contents of most amino acids (**Supplementary Table S2**; **Tables 3–5**). Obviously, the effects of *RFT1* on HD and AAC have opposite allelic directions, which is in accordance with the opposite allelic directions between QTLs for HD and PC located on the short arm of chromosome 6 (Wada et al., 2006; Yun et al., 2016).

Two other QTLs for AAC (*qAla6* and *qPro6.2*) were detected on the short of chromosome 6 in the ZS97/MY46 RIL population. They were located in the intervals RM253–RM276 and RZ667–RM19784 that are closer to the centromere region than is *RFT1*. The alleles for increasing AAC were both derived from ZS97 (**Supplementary Table S2**). At the *Hd1* locus that is tightly linked to RM19784, the functional *Hd1* ^ZS97^ allele acted to decrease HD as compared to the non-functional *Hd1* ^MY46^ allele in the ZS97 background (Zhang et al., 2012). These results suggest that the *Hd1* gene also have pleiotropic effects on HD and AAC with opposite directions.

The second and third largest QTL clusters detected in the ZS97/MY RIL population were located in the RM3325–RM3859 and RM283–RM3746 regions on the short art of chromosomes 7 and 1, covering the heading-date genes *Ghd7* (Xue et al., 2008) and *OsGI* (Hayama et al., 2003), respectively. For all the QTLs included in the two clusters, the alleles for increasing AAC were derived from ZS97 (**Supplementary Table S2**). In previous studies using the same rice cross, the ZS97 alleles in the two regions were found to decrease HD (Zhang et al., 2011; Zhang et al., 2016). These results suggest that the pleiotropic effects of major heading-date genes on AAC with opposite directions could be a common occurrence.

Similar to previous results reported by other groups (Wang et al., 2008; Lu et al., 2009; Zhong et al., 2011), our study found that it is common that a QTL region affected most components of AAC. However, it is unlikely that a gene can affect the biosynthesis of most amino acids in rice. Given that all the major QTL regions affecting AAC detected in this study were located in approximate to genes/QTLs controlling flowering time, these regions would have large effects on all traits which were influenced by heading date. It is possible that the influence of these regions on most components of AAC could be caused by indirect effects of heading date genes rather than by the direct control of these genes on the biosynthesis of amino acids. It is possible that a heading-date gene is involved in controlling nutrient transportation and accumulation in rice, either by direct involvement in the regulating network or by environmental influences on the nutrition uptake and transport due to heading date variation.

Utilization of the pleiotropic effects of heading-date genes on AAC and PC could help to meet the diverse requirements of protein for human consumption. High contents of protein and amino acids are favorable for enhancing the nutritional value, but unfavorable for eating quality (Martin and Fitzgerald, 2002; Kwon et al., 2011; Yun et al., 2016) and undesirable for some uses such as wine-making (Yoshida et al., 2002) and certain types of diet (Zhang et al., 2008). Alleles for promoting HD and increasing AAC could be selected for developing rice varieties with high nutritional values; and alleles for delaying heading and reducing AAC may be applied for developing high-yielding varieties with good eating quality. In this regard, more efforts are needed to establish a better understanding on the pleiotropism of heading-date genes on multiple traits for grain quality.

## Data Availability Statement

The raw data supporting the conclusions of this article will be made available by the authors, without undue reservation, to any qualified researcher.

## Author Contributions

J-YZ and P-SH conceived and designed the experiments. L-HX, Y-JZ, S-QT, X-JW, Z-HS, and G-AJ performed the experiments. L-HX, Y-JZ and J-YZ analysed the data. L-HX, Y-JZ and J-YZ wrote the manuscript.

## Funding

This work was funded by the National Key R&D Program of China (Grant number 2017YFD0100300), and the National Natural Science Foundation of China (Grant number 31571637).

## Conflict of Interest

The authors declare that the research was conducted in the absence of any commercial or financial relationships that could be construed as a potential conflict of interest.
