# Supplementary material for: Pleiotropic Effects of Rice Florigen Gene RFT1 on the Amino Acid Content of Unmilled Rice
Source: Front Genet. 2020 Jan 31;11:13. doi: 10.3389/fgene.2020.00013 (PMC7006618; doi:10.3389/fgene.2020.00013)
Supplement: Supplementary file 1 [file DataSheet_1.docx]

Supplementary Material

**Table S1.** Pearson correlation coefficients between 17 components of AAC in the ZS97/MY46 RIL population in 2015, 2016 and 2017

**2015**

| **Trait^a^** | **Asp** | **Thr** | **Ser** | **Glu** | **Ala** | **Gly** | **Cys** | **Val** | **Met** | **Ile** | **Leu** | **Tyr** | **Phe** | **Lys** | **His** | **Arg** |
| --- | --- | --- | --- | --- | --- | --- | --- | --- | --- | --- | --- | --- | --- | --- | --- | --- |
| Thr | 0.96^*^ |  |  |  |  |  |  |  |  |  |  |  |  |  |  |  |
| Ser | 0.95^*^ | 0.95^*^ |  |  |  |  |  |  |  |  |  |  |  |  |  |  |
| Glu | 0.91^*^ | 0.90^*^ | 0.92^*^ |  |  |  |  |  |  |  |  |  |  |  |  |  |
| Ala | 0.96^*^ | 0.92^*^ | 0.93^*^ | 0.86^*^ |  |  |  |  |  |  |  |  |  |  |  |  |
| Gly | 0.89^*^ | 0.91^*^ | 0.92^*^ | 0.90^*^ | 0.88^*^ |  |  |  |  |  |  |  |  |  |  |  |
| Cys | 0.02 | -0.02 | 0.04 | -0.02 | 0.08 | -0.01 |  |  |  |  |  |  |  |  |  |  |
| Val | 0.85^*^ | 0.89^*^ | 0.86^*^ | 0.90^*^ | 0.82^*^ | 0.89^*^ | -0.10 |  |  |  |  |  |  |  |  |  |
| Met | 0.31^*^ | 0.36^*^ | 0.34^*^ | 0.37^*^ | 0.29^*^ | 0.39^*^ | 0.03 | 0.49^*^ |  |  |  |  |  |  |  |  |
| Ile | 0.80^*^ | 0.79^*^ | 0.79^*^ | 0.83^*^ | 0.80^*^ | 0.81^*^ | 0.01 | 0.89^*^ | 0.53^*^ |  |  |  |  |  |  |  |
| Leu | 0.91^*^ | 0.86^*^ | 0.91^*^ | 0.91^*^ | 0.94^*^ | 0.87^*^ | 0.09 | 0.85^*^ | 0.32^*^ | 0.88^*^ |  |  |  |  |  |  |
| Tyr | 0.18 | 0.04 | 0.12 | 0.09 | 0.28^*^ | -0.11 | 0.27^*^ | -0.09 | -0.19 | 0.10 | 0.32^*^ |  |  |  |  |  |
| Phe | 0.81^*^ | 0.79^*^ | 0.83^*^ | 0.81^*^ | 0.84^*^ | 0.81^*^ | 0.01 | 0.77^*^ | 0.30^*^ | 0.78^*^ | 0.89^*^ | 0.24^*^ |  |  |  |  |
| Lys | 0.69^*^ | 0.64^*^ | 0.64^*^ | 0.62^*^ | 0.70^*^ | 0.58^*^ | -0.01 | 0.57^*^ | 0.13 | 0.58^*^ | 0.68^*^ | 0.31^*^ | 0.86^*^ |  |  |  |
| His | 0.74^*^ | 0.72^*^ | 0.74^*^ | 0.71^*^ | 0.78^*^ | 0.64^*^ | 0.12 | 0.60^*^ | 0.17 | 0.60^*^ | 0.75^*^ | 0.38^*^ | 0.84^*^ | 0.86^*^ |  |  |
| Arg | 0.96^*^ | 0.92^*^ | 0.93^*^ | 0.89^*^ | 0.96^*^ | 0.90^*^ | 0.05 | 0.86^*^ | 0.29^**^ | 0.83^*^ | 0.94^*^ | 0.20 | 0.83^*^ | 0.66^*^ | 0.72^*^ |  |
| Pro | 0.21 | 0.15 | 0.19 | 0.20 | 0.29^*^ | 0.04 | 0.03 | 0.04 | -0.10 | 0.17 | 0.28^*^ | 0.53^*^ | 0.29^*^ | 0.38^*^ | 0.48^*^ | 0.18 |

**2016**

| **Trait** | **Asp** | **Thr** | **Ser** | **Glu** | **Ala** | **Gly** | **Cys** | **Val** | **Met** | **Ile** | **Leu** | **Tyr** | **Phe** | **Lys** | **His** | **Arg** |
| --- | --- | --- | --- | --- | --- | --- | --- | --- | --- | --- | --- | --- | --- | --- | --- | --- |
| Thr | 0.96^*^ |  |  |  |  |  |  |  |  |  |  |  |  |  |  |  |
| Ser | 0.93^*^ | 0.94^*^ |  |  |  |  |  |  |  |  |  |  |  |  |  |  |
| Glu | 0.73^*^ | 0.68^*^ | 0.85^*^ |  |  |  |  |  |  |  |  |  |  |  |  |  |
| Ala | 0.84^*^ | 0.82^*^ | 0.82^*^ | 0.70^*^ |  |  |  |  |  |  |  |  |  |  |  |  |
| Gly | 0.88^*^ | 0.85^*^ | 0.91^*^ | 0.86^*^ | 0.83^*^ |  |  |  |  |  |  |  |  |  |  |  |
| Cys | 0.08 | -0.01 | 0.17 | 0.38^*^ | 0.05 | 0.13 |  |  |  |  |  |  |  |  |  |  |
| Val | 0.87^*^ | 0.83^*^ | 0.88^*^ | 0.85^*^ | 0.83^*^ | 0.95^*^ | 0.14 |  |  |  |  |  |  |  |  |  |
| Met | 0.29^*^ | 0.30^*^ | 0.27^*^ | 0.29^*^ | 0.31^*^ | 0.32^*^ | 0.12 | 0.39^*^ |  |  |  |  |  |  |  |  |
| Ile | 0.83^*^ | 0.79^*^ | 0.86^*^ | 0.89^*^ | 0.77^*^ | 0.95^*^ | 0.23^*^ | 0.97^*^ | 0.35^*^ |  |  |  |  |  |  |  |
| Leu | 0.84^*^ | 0.79^*^ | 0.90^*^ | 0.94^*^ | 0.79^*^ | 0.95^*^ | 0.24^*^ | 0.93^*^ | 0.34^*^ | 0.96^*^ |  |  |  |  |  |  |
| Tyr | 0.54^*^ | 0.48^*^ | 0.62^*^ | 0.69^*^ | 0.51^*^ | 0.59^*^ | 0.52^*^ | 0.58^*^ | 0.17 | 0.62^*^ | 0.65^*^ |  |  |  |  |  |
| Phe | 0.79^*^ | 0.75^*^ | 0.85^*^ | 0.87^*^ | 0.76^*^ | 0.87^*^ | 0.22 | 0.85^*^ | 0.32^*^ | 0.88^*^ | 0.94^*^ | 0.69^*^ |  |  |  |  |
| Lys | 0.81^*^ | 0.76^*^ | 0.76^*^ | 0.67^*^ | 0.82^*^ | 0.80^*^ | 0.01 | 0.81^*^ | 0.29^*^ | 0.78^*^ | 0.78^*^ | 0.55^*^ | 0.84^*^ |  |  |  |
| His | 0.85^*^ | 0.86^*^ | 0.86^*^ | 0.70^*^ | 0.85^*^ | 0.81^*^ | -0.06 | 0.80^*^ | 0.31^*^ | 0.73^*^ | 0.77^*^ | 0.46^*^ | 0.79^*^ | 0.84^*^ |  |  |
| Arg | 0.88^*^ | 0.84^*^ | 0.92^*^ | 0.86^*^ | 0.86^*^ | 0.91^*^ | 0.18 | 0.89^*^ | 0.31^*^ | 0.88^*^ | 0.91^*^ | 0.73^*^ | 0.87^*^ | 0.83^*^ | 0.85^*^ |  |
| Pro | 0.28^*^ | 0.27^*^ | 0.33^*^ | 0.42^*^ | 0.34^*^ | 0.42^*^ | 0.19 | 0.40^*^ | 0.23^*^ | 0.39^*^ | 0.39^*^ | 0.18 | 0.34^*^ | 0.33^*^ | 0.28^*^ | 0.35^*^ |

**2017**

| **Trait** | **Asp** | **Thr** | **Ser** | **Glu** | **Ala** | **Gly** | **Cys** | **Val** | **Met** | **Ile** | **Leu** | **Tyr** | **Phe** | **Lys** | **His** | **Arg** |
| --- | --- | --- | --- | --- | --- | --- | --- | --- | --- | --- | --- | --- | --- | --- | --- | --- |
| Thr | 0.95^*^ |  |  |  |  |  |  |  |  |  |  |  |  |  |  |  |
| Ser | 0.90^*^ | 0.95^*^ |  |  |  |  |  |  |  |  |  |  |  |  |  |  |
| Glu | 0.88^*^ | 0.93^*^ | 0.96^*^ |  |  |  |  |  |  |  |  |  |  |  |  |  |
| Ala | 0.10 | 0.15 | 0.14 | 0.13 |  |  |  |  |  |  |  |  |  |  |  |  |
| Gly | 0.33^*^ | 0.31^*^ | 0.34^*^ | 0.35^*^ | -0.87^*^ |  |  |  |  |  |  |  |  |  |  |  |
| Cys | 0.54^*^ | 0.59^*^ | 0.63^*^ | 0.70^*^ | 0.07 | 0.27^*^ |  |  |  |  |  |  |  |  |  |  |
| Val | 0.80^*^ | 0.83^*^ | 0.86^*^ | 0.89^*^ | 0.06 | 0.41^*^ | 0.70^*^ |  |  |  |  |  |  |  |  |  |
| Met | 0.26^*^ | 0.24^*^ | 0.31^*^ | 0.26^*^ | -0.40^*^ | 0.54^*^ | 0.18 | 0.36^*^ |  |  |  |  |  |  |  |  |
| Ile | 0.81^*^ | 0.85^*^ | 0.87^*^ | 0.91^*^ | 0.12 | 0.36^*^ | 0.66^*^ | 0.96^*^ | 0.25^*^ |  |  |  |  |  |  |  |
| Leu | 0.83^*^ | 0.87^*^ | 0.92^*^ | 0.96^*^ | 0.17 | 0.33^*^ | 0.70^*^ | 0.93^*^ | 0.26^*^ | 0.96^*^ |  |  |  |  |  |  |
| Tyr | 0.56^*^ | 0.58^*^ | 0.64^*^ | 0.68^*^ | -0.11 | 0.45^*^ | 0.81^*^ | 0.72^*^ | 0.27^*^ | 0.73^*^ | 0.73^*^ |  |  |  |  |  |
| Phe | 0.66^*^ | 0.70^*^ | 0.79^*^ | 0.81^*^ | -0.002 | 0.42^*^ | 0.71^*^ | 0.82^*^ | 0.31^*^ | 0.83^*^ | 0.88^*^ | 0.84^*^ |  |  |  |  |
| Lys | 0.78^*^ | 0.79^*^ | 0.75^*^ | 0.76^*^ | 0.03 | 0.38^*^ | 0.55^*^ | 0.80^*^ | 0.24^*^ | 0.82^*^ | 0.81^*^ | 0.64^*^ | 0.72^*^ |  |  |  |
| His | 0.75^*^ | 0.78^*^ | 0.79^*^ | 0.81^*^ | 0.19 | 0.24^*^ | 0.63^*^ | 0.81^*^ | 0.17 | 0.80^*^ | 0.83^*^ | 0.63^*^ | 0.74^*^ | 0.84^*^ |  |  |
| Arg | 0.81^*^ | 0.85^*^ | 0.88^*^ | 0.89^*^ | 0.11 | 0.37^*^ | 0.68^*^ | 0.92^*^ | 0.33^*^ | 0.90^*^ | 0.91^*^ | 0.74^*^ | 0.80^*^ | 0.80^*^ | 0.88^*^ |  |
| Pro | 0.43^*^ | 0.42^*^ | 0.44^*^ | 0.46^*^ | -0.73^*^ | 0.92^*^ | 0.34^*^ | 0.49^*^ | 0.48^*^ | 0.44^*^ | 0.41^*^ | 0.50^*^ | 0.48^*^ | 0.41^*^ | 0.30^*^ | 0.45^*^ |

^a^ The contents of the amino acids are presented as % in brown rice. Asp, Aspartic acid; Thr, Threonine; Ser,Serine; Glu, Glutamic Acid; Gly, Glycine; Ala, Alanine; Cys, Cystine; Val, Valine; Met, Methionine; Ile, Isoleucine; Leu, Leucine; Tyr, Tyrosine; Phe, Phenylalanine; Lys, Lysine; His, Histidine; Arg, Arginine and Pro, Proline.

^*^*P* < 0.003

**Table S2.** QTLs for 17 components of AAC detected in the ZS97/MY46 RIL population.

| **Trait** | **QTL^a^** | **Interval** | **2015** | | |  | **2016** | | |  | **2017** | | |
| --- | --- | --- | --- | --- | --- | --- | --- | --- | --- | --- | --- | --- | --- |
|  |  |  | ***LOD*** | ***A*^b^** | ***R^2^*(%)^c^** |  | **LOD** | ***A*** | ***R^2^*(%)** |  | ***LOD*** | ***A*** | ***R^2^*(%)** |

Asp *qAsp1* RG146-RM24 - - - - - - 3.41 -0.023 17.0

*qAsp6* RM225-RM6917 3.77 0.029 8.1 3.01 0.021 7.3 3.08 0.024 17.0

*qAsp7* RM3325-RM1243 3.53 -0.030 8.7 - - - 2.50 -0.025 19.8

Thr *qThr2* RM263-RM6 2.28 0.011 6.3 - - - - - -

*qThr4* RG776A-RG620 2.04 -0.009 4.3 - - - - - -

*qThr6* RM225-RM6917 3.64 0.012 7.6 3.52 0.009 8.3 - - -

*qThr7* RM3325-RM1243 2.32 -0.010 5.8 - - - - - -

*qThr11* RM287-RM209 2.93 -0.011 6.5 2.29 0.008 5.2 - - -

Ser *qSer1* RG532-RM151 - - - 2.14 -0.008 4.9 - - -

*qSer2* RM6-RM240 2.34 0.015 5.8 - - - - - -

*qSer6* RM225-RM6917 4.42 0.022 9.6 2.36 0.010 6.1 4.33 0.013 3.8

*qSer7* RM1243-RM3859 - - - - - - 2.01 -0.011 4.3

*qSer8* RG108-RIX4 - - - - - - 2.09 -0.009 3.6

Glu *qGlu3* RZ328-RZ575 - - - 2.13 0.042 5.8 - - -

*qGlu6* RM225-RM6917 6.86 0.100 15.4 - - - 2.90 0.037 5.2

*qGlu11* RZ816-RM332 2.28 -0.005 5.5 - - - - - -

Gly *qGly1* RM294A-RM294B - - - - - - 2.60 -0.027 32.8

*qGly4* RM3317-RM401 2.01 -0.006 4.4 - - - - - -

*qGly6* RM225-RM6917 2.33 0.006 4.8 2.33 0.005 5.6 3.13 0.032 33.9

*qGly7.1* RM3325-RM1243 2.90 -0.008 7.9 - - - - - -

*qGly7.2* RM182-RM336 - - - - - - 3.44 0.032 35.9

*qGly11* RM287-RM209 2.08 -0.006 5.0 - - - - - -

Ala *qAla6* RM253-RM276 - - - - - - 2.10 -0.025 23.3

*qAla7* RM3325-RM1243 3.96 -0.009 10.9 - - - - - -

Cys *qCys7* RZ721-RZ395 - - - - - - 3.35 0.010 16.5

*qCys9* RM105-RM3700 2.28 0.026 6.0 - - - - - -

*qCys12* RG81-S13126 - - - - - - 4.06 0.011 15.3

Val *qVal1* RG532-RM151 - - - - - - 2.50 -0.008 7.5

*qVal2* RM6-RM240 2.05 0.018 7.8 - - - - - -

*qVal4* RM303-RG214 2.27 -0.014 5.3 - - - - - -

*qVal6* RM225-RM6917 3.61 0.018 7.7 - - - 2.24 0.008 7.3

*qVal7* RM3325-RM1243 2.60 -0.016 6.6 - - - - - -

Met *qMet6* RM190- RZ516 2.92 0.008 7.2 - - - 2.15 0.006 17.2

*qMet9* RZ698-RM296 - - - - - - 2.35 -0.008 16.2

*qMet11* RZ816-RM332 - - - 2.25 0.007 5.6 - - -

Ile *qIle1* RG532-RM151 - - - - - - 2.89 -0.006 9.7

*qIle2* RM240-RZ123 2.48 0.011 6.9 - - - - - -

*qIle4* RM3317-RM401 - - - - - - 2.00 -0.005 9.1

*qIle11* RG167-RM287 - - - - - - 2.50 -0.006 10.1

Leu *qLeu1* RG532-RM151 - - - - - - 2.88 -0.016 15.0

*qLeu6* RM6917-RZ450 3.26 0.030 9.4 2.15 0.016 5.3 2.61 0.014 13.7

Tyr *qTyr1* RM151-RM3746 - - - - - - 2.34 -0.007 12.3

*qTyr2* RZ318-RM263 - - - 3.24 -0.010 9.5 - - -

*qTyr6* RZ398- RM204 2.83 0.019 6.5 - - - 2.28 0.007 11.3

*qTyr7.1* RM1243-RM3859 - - - - - - 2.96 -0.009 13.4

*qTyr7.2* RZ626-RG650 - - - - - - 3.76 -0.010 10.7

*qTyr12* RM20-RG81 2.62 -0.022 9.1 - - - - - -

^a^QTLs are designated as proposed by McCouch and CGSNL (2008).

^b^Additive effect of replacing a ZS97 allele with a MY46 allele.

^c^Proportion of phenotypic variance explained by the QTL effect.

-, not significant.

**Table S2.** Continued.

| **Trait** | **QTL^a^** | **Interval** | **2015** | | |  | **2016** | | |  | **2017** | | |
| --- | --- | --- | --- | --- | --- | --- | --- | --- | --- | --- | --- | --- | --- |
|  |  |  | ***LOD*** | ***A*^b^** | ***R^2^*(%)^c^** |  | **LOD** | ***A*** | ***R^2^*(%)** |  | ***LOD*** | ***A*** | ***R^2^*(%)** |

Phe *qPhe4* RM303-RG214 2.78 -0.020 6.4 - - - - - -

*qPhe6* RM225-RM6917 2.62 0.019 5.6 2.45 0.013 6.3 2.53 0.014 24.6

*qPhe7* RM3325-RM1243 2.51 -0.020 7.0 - - - - - -

*qPhe9* RM257-RM242 2.96 -0.020 6.6 - - - - - -

Lys *qLys1.1* RG532-RM151 - - - - - - 2.65 -0.007 9.3

*qLys1.2* RG381-RG236 2.58 -0.014 8.0 - - - - - -

*qLys6* RM225-RM6917 - - - - - - 2.68 0.008 10.1

*qLys9* RM242-RM108 2.87 -0.013 6.6 - - - - - -

*qLys12* RM20-RG81 3.24 -0.017 10.8 - - - - - -

His *qHis1* RG532-RM151 - - - 2.15 -0.005 4.9 - - -

*qHis2* RZ123-RM208 - - - 2.54 0.006 7.1 - - -

*qHis4* RG454-RM273 3.91 -0.013 8.5 - - - - - -

*qHis6* RZ398- RM225 2.53 0.011 4.9 3.24 0.006 7.4 5.04 0.006 5.2

*qHis9* RM242-RM108 3.15 -0.012 6.9 - - - - - -

*qHis12* RM20-RG81 2.68 -0.012 6.9 - - - - - -

Arg *qArg3* RZ328-RZ575 - - - 2.38 0.016 6.6 - - -

*qArg6* RM225-RM6917 2.22 0.017 4.7 2.27 0.014 5.5 3.92 0.019 2.6

*qArg7* RM3325-RM1243 3.91 -0.020 9.9 - - - 2.21 -0.017 2.2

Pro *qPro1.1* RM283-RG532 2.35 -0.022 5.7 - - - - - -

*qPro1.2* RM24-RM294A - - - - - - 3.00 -0.023 20.9

*qPro1.3* RM237-RM246 - - - 2.12 0.018 5.8 - - -

*qPro6.1* RM225-RM6917 - - - - - - 2.50 0.020 20.4

*qPro6.2* RZ667-RM19784 2.38 -0.020 7.0 - - - - - -

*qPro7.1* RZ471-RM320 - - - - - - 5.00 -0.036 21.7

*qPro7.2* RM336-RZ264 - - - - - - 2.58 0.025 20.0

*qPro10* RM5348-RM1859 - - - 2.32 0.023 7.0 - - -

^a^QTLs are designated as proposed by McCouch and CGSNL (2008).

^b^Additive effect of replacing a maternal allele by a paternal allele.

^c^Proportion of phenotypic variance explained by the QTL effect.

-, not significant.
